# Fine Tuning of Redox Networks on Multiheme Cytochromes from *Geobacter sulfurreducens* Drives Physiological Electron/Proton Energy Transduction

**DOI:** 10.1155/2012/298739

**Published:** 2012-07-31

**Authors:** Leonor Morgado, Joana M. Dantas, Marta Bruix, Yuri Y. Londer, Carlos A. Salgueiro

**Affiliations:** ^1^Requimte-CQFB, Departamento de Química, Faculdade de Ciências e Tecnologia, Universidade Nova de Lisboa, Campus Caparica, 2829-516 Caparica, Portugal; ^2^Departamento de Espectroscopía y Estructura Molecular, Instituto de Química-Física “Rocasolano”, CSIC, Serrano 119, 28006 Madrid, Spain; ^3^Biosciences Division, Argonne National Laboratory, Argonne, IL 60439, USA; ^4^New England Biolabs, 240 County Road, Ipswich, MA 01938, USA

## Abstract

The bacterium *Geobacter sulfurreducens (Gs)* can grow in the presence of extracellular terminal acceptors, a property that is currently explored to harvest electricity from aquatic sediments and waste organic matter into microbial fuel cells. A family composed of five triheme cytochromes (PpcA-E) was identified in *Gs*. These cytochromes play a crucial role by bridging the electron transfer from oxidation of cytoplasmic donors to the cell exterior and assisting the reduction of extracellular terminal acceptors. The detailed thermodynamic characterization of such proteins showed that PpcA and PpcD have an important redox-Bohr effect that might implicate these proteins in the e^−^/H^+^ coupling mechanisms to sustain cellular growth. The physiological relevance of the redox-Bohr effect in these proteins was studied by determining the fractional contribution of each individual redox-microstate at different pH values. For both proteins, oxidation progresses from a particular protonated microstate to a particular deprotonated one, over specific pH ranges. The preferred e^−^/H^+^ transfer pathway established by the selected microstates indicates that both proteins are functionally designed to couple e^−^/H^+^ transfer at the physiological pH range for cellular growth.

## 1. Introduction

The ability to use extracellular terminal electron acceptors (e.g., Fe(III), U(VI) oxides, or electrode surfaces) in addition to the more common cytoplasmic acceptors, such as fumarate, spreads the bacterium *Geobacter sulfurreducens* (*Gs*) environmental versatility [1–3]. However, the use of extracellular acceptors sets to the microorganism additional challenges. The first of such is the efficient electron delivery to cell exterior, and the second one is the net production of metabolic energy to support cellular growth. To address them, *Gs* respiratory chains are designed to permit an effective flow of electrons from the oxidation of cytoplasmic organic compounds to the outer membrane. In fact, the topology of the electron transfer proteins involved in such electron transfer is quite unusual in comparison with other Gram-negative bacteria. In addition to the localization at the cytoplasmic membrane, several electron transfer proteins have also been identified at the outer membrane of *Gs* cells, which constitutes an efficient interface between the cell surface and the extracellular acceptors [4–8]. Albeit much remains to be known on the *Geobacter*'s electron transfer chains, it is consensual nowadays that electrons are transferred at the cytoplasmic membrane from a NAD(P)H dehydrogenase to a quinone pool, then to periplasmic *c*-type cytochromes, and, finally, to at least a metal reductase at the outer membrane [9]. The use of extracellular electron acceptors by *Gs* leads to a decrease in the biomass production in comparison with the soluble acceptor fumarate [10]. The discrepancy observed in the cellular growth yield was attributed to the cytoplasmic acidification, which is circumvented when the acceptor is fumarate. In this case, the cytoplasmic protons produced from acetate oxidation are consumed favouring the H^+^ electrochemical potential gradient across the periplasmic membrane that drives ATP synthesis [11]. In comparison to the studies on fumarate respiration, metabolic modelling studies can only simulate the experimental results obtained in presence of extracellular electron acceptors if additional e^−^/H^+^ coupling mechanisms are considered [10]. To date, these mechanisms remain to be identified, and the present work aims to contribute to their clarification.

Evidence for functional energy transduction in the absence of a membrane confinement was reported for the hydrogenase/tetraheme cytochrome *c*_3_ system from *Desulfovibrio vulgaris* [12, 13]. In this case, the electrons and protons from H_2_ are provided by the periplasmic hydrogenase to the periplasmic cytochrome *c*_3_, which couples the deenergization of electrons to the lowering of proton *pK*_*a*_ values to favour their release in the periplasm. This hydrogenase/cytochrome *c*_3_ system works as a proton thruster device, which provides the required diffusion control and thermodynamic drive in the water-protein interface (a two-phase system) in agreement with the Williams localized theory [14].

A family composed of five triheme cytochromes (PpcA-E) was identified in *Gs *[15]. These five cytochromes are small soluble proteins, each with approximately 10 kDa. Cytochromes PpcB, PpcC, PpcD, and PpcE share 77%, 62%, 57%, and 65% amino acid sequence identity with PpcA, respectively [15]. The three hemes form the protein heme core and are covalently linked to cysteine residues in the CXXCH binding motifs, where X designates any amino acid. All the hemes are axially coordinated by two histidine residues and are low spin, both in the reduced (Fe^2+^, S = 0) and in the oxidized (Fe^3+^, S = 1/2) forms. The structures of the five cytochromes have been determined, showing that they have a high level of structural homology [16, 17]. Knockout studies on *Gs* cells with the genes encoding PpcA and PpcD deleted showed that cellular Fe^3+^ reduction was impaired [18]. In addition, proteomics studies by Ding and coworkers [19] showed that PpcD is more abundant when *Gs* is grown on insoluble iron oxides compared to ferric citrate. Like cytochromes *c*_3_, PpcA and PpcD can be reduced by hydrogenase and their thermodynamic characterization showed that they have the adequate properties to couple e^−^/H^+^ transfer, which might contribute for the H^+^ electrochemical potential gradient necessary to support bacterial growth in presence of extracellular acceptors [20]. 

In this work, we show that the e^−^/H^+^ coupling in PpcA and PpcD driven by the protonation/deprotonation of the redox-Bohr center is observed only within the cellular optimal pH range for growth, reinforcing the physiologically significance of the redox-Bohr effect observed for these bacteria. 

## 2. Methodology

**Figure 1:**
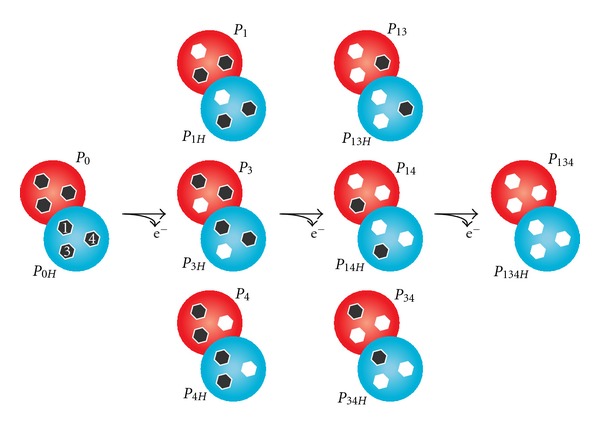
Electronic distribution scheme for a triheme cytochrome with a proton-linked equilibrium showing the 16 possible microstates. PpcA and PpcD are structurally similar to tetraheme cytochromes *c*_3_, with the exception that heme 2 and the corresponding fragment of the polypeptide chain are absent. Thus, to be consistent with the literature, the heme groups are numbered 1, 3, and 4 according to the order of attachment to the CXXCH motif in the polypeptide chain. The blue and red circles correspond to the protonated and deprotonated microstates, respectively. Hexagons represent heme groups, which can be either reduced (black) or oxidized (white). The microstates are grouped, according to the number of oxidized hemes, in four oxidation stages connected by three one-electron redox steps. *P*_0*H*_ and *P*_0_ represent the reduced protonated and deprotonated microstates, respectively. *P*_*ijkH*_ and *P*_*ijk*_ indicate, respectively, the protonated and deprotonated microstates, where *i*, *j*, and *k* represent the heme(s) that are oxidized in that particular microstate.

The coexistence of several microstates in solution, connecting the fully reduced and oxidized states, makes the study of the properties of the redox centers in multiheme proteins particularly complex. In the case of *Gs* triheme cytochromes, a single *pK*_*a*_ dominates the pH dependence of the reduction potentials of the heme groups [20], and thus 16 possible microstates can coexist in solution (Figure 1). The several microstates can be grouped in four macroscopic oxidation stages (*S*_0−3_) connected by three one-electron redox steps and containing the microstates with the same number of oxidized hemes. Each pair of microstates (protonated or deprotonated) is connected by single electron redox steps and can be described by a total of 24 Nernst equations. In each case, three Nernst equations relate the microstates in oxidation stage 1 to that of stage 0; six equations relate microstates in stage 2 and 1; finally, three equations describe the redox equilibria between the fully oxidized microstate and those in stage 2. Taking as reference the fully reduced and protonated microstate and considering a sequential oxidation of hemes 1, 3, and 4 along the four oxidation stages as an example, the relevant Nernst equations are:

(1) $$\begin{array}{c} E=e_{1}+\frac{RT}{F}\;\ln\;\frac{P_{1H}}{P_{0H}},\\ E=e_{3}+\;I_{13}+\frac{RT}{F}\;\ln\;\frac{P_{13H}}{P_{1H}},\\ E=e_{4}+\;I_{13}+I_{14}{+I}_{34}+\frac{RT}{F}\;\ln\;\frac{P_{134H}}{P_{13H}}, \end{array}$$

where the term *e*_*i*_ represents the heme reduction potentials and *I*_*ij*_ the heme redox interactions, which account for the effect of the oxidation state of one heme in the reduction potential of its neighbors. 

These equations (1) can be rewritten and each microstate can be expressed as a function of *P*_0*H*_ (2):

(2) $$\begin{array}{c} P_{1H}=P_{0H}\;\exp^{(E-e_{1})F/RT},\\ P_{13H}=P_{0H}\;\exp^{(2E-e_{1}-e_{3}-I_{13})F/RT},\\ P_{134H}=P_{0H}\;\exp^{(3E-e_{1}-e_{3}-e_{4}-I_{13}-I_{14}-I_{34})F/RT}. \end{array}$$

The fully reduced and protonated microstate *P*_0*H*_ and the correspondent deprotonated microstate *P*_0_ are related by the Handerson-Hasselbach equation:

(3) $$\begin{array}{c} P_{0H}⇄P_{0}+{\text{H}}^{+},\\ P_{0}=P_{0H}\;\exp^{\left[\ln10(pH-{pK}_{\text{red}})\right]}. \end{array}$$

Taking as an example the sequential oxidation of hemes 1, 3, and 4 along the four oxidation stages but for the deprotonated microstates, the Nernst equations are:

(4) $$\begin{array}{c} {E=e}_{1}+\;I_{1H}+\frac{RT}{F}\;\ln\;\frac{P_{1}}{P_{0}}\\ {E=e}_{3}+\;I_{13}+\;I_{1H}+\;I_{3H}+\frac{RT}{F}\;\ln\;\frac{P_{13}}{P_{1}}\\ {E=e}_{4}+\;I_{13}+I_{14}{+I}_{34}+\;I_{1H}+I_{3H}+I_{4H}+\frac{RT}{F}\;\ln\;\frac{P_{134}}{P_{13}}, \end{array}$$

where the terms *I*_*iH*_ represent the redox-Bohr interactions and account for the effect of the pH on the heme reduction potentials. Equation (4) can also be rewritten by expressing each deprotonated microstate as a function of *P*_0*H*_ (5):

(5) $$\begin{array}{c} P_{1}=P_{0H}\;\exp^{\left[\left(\ln10(pH-{pK}_{\text{red}}))+(\left(E-e_{1}-I_{1H})(F/RT\right)\right)\right]}\\ P_{13}=P_{0H}\;\exp^{\left[\left(\ln10(pH-{pK}_{\text{red}}))+(\left(2E-e_{1}-e_{3}-I_{13}-I_{1H}-I_{3H})(F/RT\right)\right)\right]}\\ P_{134}=P_{0H}\;\exp^{\left[\left(\ln10(pH-{pK}_{\text{red}}))+(\left(3E-e_{1}-e_{3}-e_{4}-I_{13}-I_{14}-I_{34}-I_{1H}-I_{3H}-I_{4H})(F/RT\right)\right)\right]}. \end{array}$$

Thus, in the particular case of a triheme cytochrome, three reduction potentials, one *pK*_red_ plus six two-center interactions (three heme and three redox-Bohr interactions), are sufficient to determine the fractional contribution of each microstate, across the full range of pH and solution potential. 

These parameters can be obtained combining data from NMR and visible spectroscopy and were determined for PpcA and PpcD in a previous work (Table 1) [20].

## 3. Results and Discussion 

In this work, the effect of pH on the functional mechanism of the triheme cytochromes PpcA and PpcD from *Gs* was evaluated. The thermodynamic parameters listed on Table 1 for the fully reduced and protonated proteins, which include the heme reduction potentials, the *pK*_*a*_ values of the redox-Bohr center, heme-heme redox interactions, and redox-Bohr interactions, were used to determine the heme oxidation profiles and the fractional contribution of the 16 microstates at different pH values. This allows to compare the individual heme oxidation profiles inside and outside the physiological pH range and to demonstrate a correlation between the functional properties of these cytochromes and the physiological pH range for cellular growth.

From the heme reduction potential values listed in Table 1, the order of oxidation of the heme groups can be established for the fully reduced and protonated protein, which is 1-3-4 for PpcA and 1-4-3 for PpcD. However, both proteins display important redox-Bohr interactions, being the largest one observed for heme 4. All the redox-Bohr interactions are negative, which is expected in electrostatic terms, since the removal of proton(s), upon deprotonation of the redox-Bohr center, lowers the affinity for electrons (lower reduction potential) by the heme groups. This is reflected in the heme reduction potentials of the fully reduced deprotonated proteins, which can be obtained by the simple sum of the heme reduction potentials of the fully reduced and protonated proteins with their respective redox-Bohr interactions. In the case of PpcA, the heme reduction potentials for the fully reduced and deprotonated protein are −186, −169, and −183 mV for hemes 1, 3, and 4, respectively. Comparison of these values with those obtained for the correspondent protonated form (Table 1) clearly shows that the order of oxidation is different in both situations: 1-4-3 (deprotonated protein) and 1-3-4 (protonated protein). For PpcD, the same scenario is observed: the heme reduction potentials of the reduced and deprotonated protein are more negative (−184, −162, and −202 mV for hemes 1, 3, and 4, resp.), and the order of oxidation is affected by the deprotonation of the redox-Bohr center (4-1-3 *versus* 1-4-3). This analysis shows that the redox properties of PpcA and PpcD are modulated by the solution pH (redox-Bohr effect). However, the redox-Bohr effect is functionally relevant only if observed at physiological pH range for cellular growth. 

To best of our knowledge, the optimal pH for *Gs* growth has not yet been determined. Kim and Lee [21] studied the effect of the initial pH on the growth rates of *Gs* cultures utilizing fumarate as electron acceptor in the pH range of 5.5–6.8. Within this range, higher rates were observed at pH 6.8, which were reduced at pH 6.4 and completely inhibited at pH 5.5 [21]. In addition to this non-electrode-respiring conditions, several electrode-respiring studies have shown a pH drop inside *Gs* biofilms with a concomitant decrease in the measured current [22–25]. Experiments carried out with anode-respiring bacteria with high presence of *Gs* cells have shown that in the pH range 6–8, maximum current density was achieved at pH 8 and dropped continually to pH 6 [22–24]. These studies also suggested that at pH < 6 *Gs* metabolism is inhibited.

**Figure 2:**
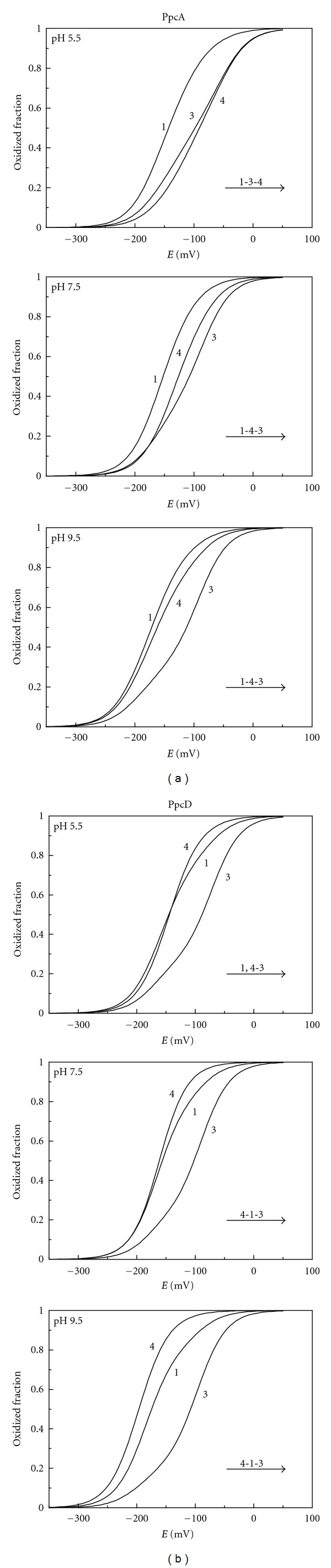
Heme oxidation fractions for PpcA (a) and PpcD (b) at different pH values. The curves were calculated as a function of the solution reduction potential (*versus* SHE) using the parameters listed in Table 1. The order of oxidation of the hemes is indicated by the arrow.

**Figure 3:**
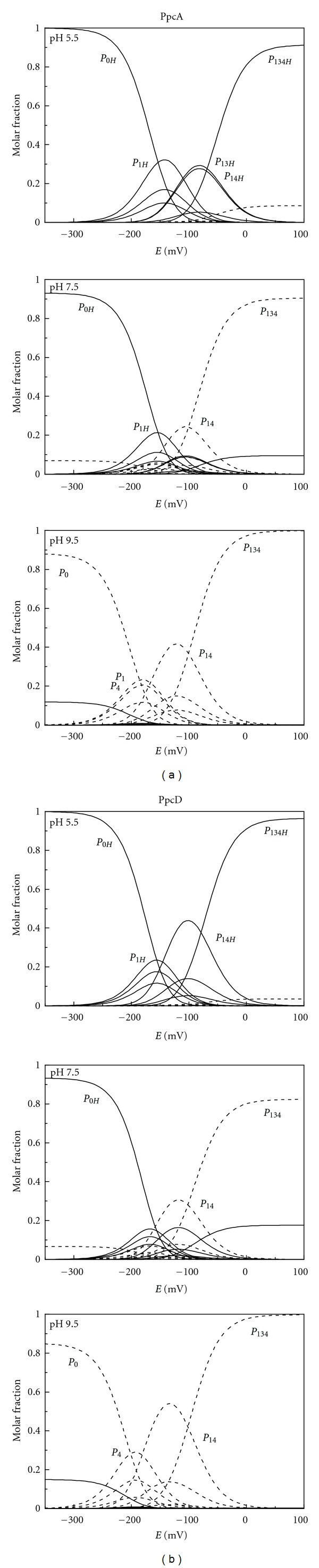
Molar fraction of the 16 individual microstates (see Figure 1) of PpcA (a) and PpcD (b) at different pH values. The curves were calculated as a function of the solution reduction potential (*versus* SHE) using the parameters listed in Table 1. Solid and dashed lines indicate the protonated and deprotonated microstates, respectively. For clarity, only the relevant microstates are labeled.

**Figure 4:**
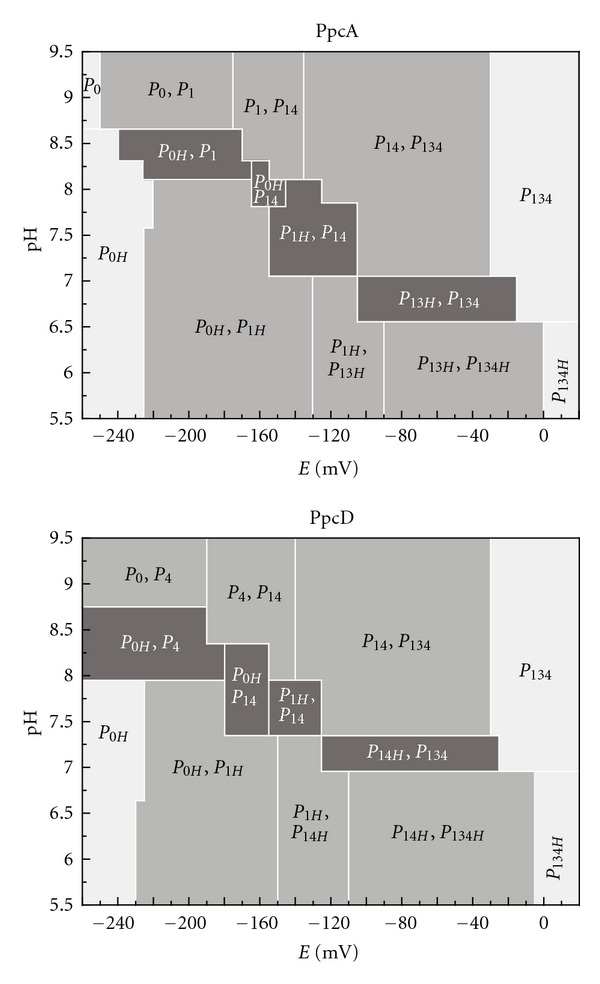
Dependence of the molar fractions of PpcA and PpcD microstates with pH and solution potential (*versus* SHE) at 288 K and 250 mM ionic strength. The molar fractions of the individual microstates were determined using the parameters listed in Table 1, and those showing the largest molar fraction are represented. The regions where proteins can perform e^−^/H^+^ transfer and e^−^ transfer are highlighted in dark gray and gray, respectively. Regions where the protein is fully reduced or fully oxidized are indicated in light gray.

In a previous work, we have used pH 7.5 as representative value for *Gs* physiological pH [20]. In the present work, we aimed to evaluate the significance of the redox-Bohr effect on the functional mechanism of PpcA and PpcD by studying the individual heme oxidation profiles and the fractional contribution of the microstates at a broader pH range. Thus, in order not to confuse literature, in the present work, the individual heme oxidation profiles and fractional contribution of microstates of both proteins were determined at pH 5.5 and 9.5 and compared with the previous analysis carried out at pH 7.5 (Figures 2 and 3). Then, a detailed analysis of the dominant PpcA and PpcD microstates was carried out in the pH range 5.5–9.5 (Figure 4).

The heme oxidation profiles described in Figure 2 show that, at each pH value, the shape of the redox curves is substantially different from a pure Nernst curve and the several crossovers clearly indicate that the electron affinity of each heme is modulated by the heme-heme redox interactions, as protein oxidation progresses. The redox interactions are all positive, which is expected in electrostatic terms, and reflect the stabilization of the reduced state of one heme by the removal of one electron from a neighboring one. From the comparison of the individual heme oxidation profiles at different pH values, it is clear that the heme apparent midpoint reduction potentials *e*_app_ (i.e., the point at which the oxidized and reduced fractions of each heme group are equally populated) are different due to redox-Bohr interactions (Figure 2). The shape of each heme oxidation curve is therefore a result of the interplay of both heme-heme and redox-Bohr interactions. 

In the case of PpcA, at pH 5.5, the *e*_app_ values of hemes 3 and 4 are similar. However, at high pH, the deprotonation of the redox-Bohr center lowers considerable the *e*_app_ value of heme 4 (largest redox-Bohr interaction) bringing it closer to that of heme 1. The modulation of the individual heme oxidation profiles is also observed for PpcD, though yielding a distinct result. In fact, due to the similarity of the *e*_app_ values of hemes 1 and 4 at low pH, the deprotonation of the redox-Bohr center yields a more notorious separation of the three curves being heme 4 the one with smaller *e*_app_ value. 

The effect of the protonation/deprotonation of the redox-Bohr center can be further rationalised from the fractional contribution of each microstate (Figure 1). The fractional contribution of these microstates for PpcA and PpcD at pH 5.5, 7.5, and 9.5 provides functional mechanistic insights on the electron transfer pathways of the proteins (Figure 3). The analysis of this figure shows that the relevant microstates are quite distinct at different pH values. In the case of PpcA, several microstates dominate the intermediate stages of oxidation either at pH 5.5 or pH 9.5. At pH 7.5, stage 0 is dominated by the protonated form *P*_0*H*_ and stage 1 is dominated by the oxidation of heme 1 (*P*_1*H*_) while keeping the acid-base center protonated. Stage 2 is dominated by the oxidation of heme 4 and deprotonation of the acid-base center (*P*_14_), which remains deprotonated in stage 3 (*P*_134_). Therefore, at pH 7.5, a route is defined for the electrons within PpcA: *P*_0*H*_ → *P*_1*H*_ → *P*_14_ → *P*_134_, whereas at the other pH values there is no coherent path. Moreover, it is also clear that, associated with the favoured electron transfer pathway at physiologic pH, a deprotonation occurs as one electron is transferred between oxidation stages 1 and 2 (Figure 3) suggesting that microstates *P*_14_ and *P*_1*H*_ are the physiological forms of PpcA. This mechanistic information, which can only be obtained from a detailed microscopic analysis shows how selected microstates could confer directionality of events: PpcA microstate *P*_14_ can uptake electrons and a weakly acidic proton from the donor associated with the cytoplasmic membrane, originating the microstate *P*_1*H*_. The *pK*_*a*_ value of this proton (*pK*_*a*_ 8) corresponds to the *pK*_*a*_ value of oxidation stage 1 (Table 1). As discussed above, upon protonation of the redox-Bohr center, the reduction potential of the hemes becomes less negative. The loss of electron reducing power is used to lower the *pK*_*a*_ of the proton so that when meeting the physiological downstream redox partner, microstate *P*_1*H*_ donates less reducing electrons and a more acidic proton (*pK*_*a*_ 7.2, which corresponds to the *pK*_*a*_ value of oxidation stage 2—see Table 1). This is now sufficiently acidic to be released in the periplasm, and microstate *P*_14_ is now ready to initiate a new energy transduction cycle.

PpcD can also couple the transfer of electrons and protons at physiologic pH, though by a different pathway (Figure 3). The oxidation stage 0 is dominated by the protonated form *P*_0*H*_. However, the microstates of oxidation stage 1 are overcomed by the *P*_0*H*_ curve, which intercepts first the *P*_14_ curve. This microstate (*P*_14_) dominates the oxidation stage 2, whereas *P*_134_ dominates stage 3. Thus, for this cytochrome, a different preferential route for electrons is established, favouring a proton-coupled 2 e^−^ transfer step between oxidation stages 0 and 2: *P*_0*H*_ → *P*_14_ → *P*_134_. As for PpcA, at pH 5.5 and 9.5, no coupling between electron and proton transfer is observable for PpcD (Figure 3).

Several studies using biofilms on electrodes have shown that electron transfer out of *Gs* cells is detectable above potentials of about −200 mV *versus* standard hydrogen electrode [22, 26, 27]. Thus, in the present work, we further determined the microstates of PpcA and PpcD showing the largest molar fraction values in the redox potential window −200 mV to −30 mV and also in the pH range 5.5–9.5 (Figure 4). This analysis shows that PpcA and PpcD are not fully reduced nor fully oxidized and thus are functionally active (i.e., capable of receiving and donating electrons) in that redox potential and pH range. Additionally, it is interesting to note that the microstates pairs that can couple e^−^/H^+^ transfer are essentially detectable at the pH range for which most studies with *Geobacter* cells have been carried out. In particular, PpcA, which is highly abundant in *Gs*, being most likely the reservoir of electrons destined for outer surface [28, 29], can perform e^−^/H^+^ energy transduction in the pH range 6.5 to 8.5 albeit at different redox potential values (Figure 4). It should be emphasized that the data presented in Figure 4 was obtained for purified proteins and that the transposition of these data to *Gs* cells grown in cultures or biofilms should take into account that in these more complex systems, other variables such as the presence of the other cellular components, metabolic status, and environmental conditions (e.g., ionic strength) can alter slightly the working range of these proteins. Studies on *Gs* biofilms on electrodes showed that cells have a Nernstian response around −150 mV [26, 30, 31]. Since PpcA is functionally active at this redox potential (Figure 4), it can be hypothesized that this response is mainly PpcA driven. Under this hypothesis and since PpcA is also able to perform e^−^/H^+^ energy transduction at pH values around 7, a possible functional mechanism would involve the transfer of one electron and one proton from the quinone oxidoreductase being the second electron transfer back to the quinone pool.

Overall, the work presented here shows that PpcA and PpcD display the adequate functional properties to perform e^−^/H^+^ energy transduction but only within the pH range 6.5–8.5. This might represent additional mechanisms contributing to the H^+^ electrochemical potential gradient across the periplasmic membrane that drives ATP synthesis and might also explain why *Gs* cells become metabolically inactive at pH 6 or lower [22–25].

## Figures and Tables

**Table 1:** Thermodynamic parameters of the fully reduced and protonated forms of PpcA and PpcD obtained at 288 K and 250 mM ionic strength [20]. Redox potentials are relative to standard hydrogen electrode (SHE). Standard errors are given in parenthesis. The *pK*_1_, *pK*_2_, and *pK*_ox_ values were determined from the values obtained for *pK*_red_ and redox-Bohr interactions.

		PpcA	PpcD
Heme redox potentials (mV)	*e*_1_	−154(5)	−156(6)
	*e*_3_	−138(5)	−139(6)
	*e*_4_	−125(5)	−149(6)

Heme-heme redox interactions (mV)	*I*_13_	27(2)	46(3)
	*I*_14_	16(3)	3(4)
	*I*_34_	41(3)	14(4)

Redox-Bohr interactions (mV)	*I*_1*H*_	−32(4)	−28(6)
	*I*_3*H*_	−31(4)	−23(6)
	*I*_4*H*_	−58(4)	−53(6)

*pK*_*a*_	Stage 0 (*pK*_red_)	8.6 (0.1)	8.7 (0.1)
	Stage 1 (*pK*_1_)	8.0 (0.1)	8.1 (0.1)
	Stage 2 (*pK*_2_)	7.2 (0.1)	7.4 (0.1)
	Stage 3 (*pK*_ox_)	6.5 (0.1)	6.9 (0.1)

## Abbreviations

*Gs*: *Geobacter sulfurreducens* bacterium
Ppc: Periplasmic *c*-type cytochrome
SHE: Standard hydrogen electrode.
